# Supplementary material for: Quorum Sensing Signaling Molecules Positively Regulate c-di-GMP Effector PelD Encoding Gene and PEL Exopolysaccharide Biosynthesis in Extremophile Bacterium Acidithiobacillus thiooxidans
Source: Genes (Basel). 2021 Jan 7;12(1):69. doi: 10.3390/genes12010069 (PMC7825692; doi:10.3390/genes12010069)
Supplement: Supplementary file 1 [file genes-12-00069-s001.pdf]

Supplementary material

Table S1. Summary results of fluorescence binding analysis obtained with twelve different lectins in *A. thiooxidans* wild type cells.

Lectin	Specificity[a]	*A. thiooxidans*[T]
**AAL**	L-Fucose α(1,6) N-Acetyl-D-Glucosamine	+
**BPA**	N-Acetyl-D-Galactosamine	+
**ConA**	Internal D-Mannose and D-Glucose	+
**DBA**	Terminal α-N-Acetyl-D-Galactosamine	-
**GS-I**	α-D-Galactose	-
**GS-II**	N-Acetyl-D-Glucosamine	+
**LPA**	N-acetylneuraminic acid	-
**MPA**	α-D-Galactose	-
**PNA**	Galactoseβ(1,4)Glucose	-
**SBA**	Galactose	-
**UEA-I**	Linked α(1,2) L-Fucose	-
**WGA**	GlcNacβ(1,4) GlcNacβ(1,4) GlcNac	-

[a], according to the manufacturer´s information (EY Laboratories®, San Mateo, CA, USA).

Table S2. Primers used in this work.

Name	Sequence 5′–3′ [1]
*pelA1*_F	CCGATTGCCGCAGTTATTTATT
*pelA1*_R	GCTGTCTTGATGGCTTTGATG
*pelD*_F	CACAAGTTGGCATCCTGGTTCGTT
*pelD*_R	CATGCTGCCTGCGAAAGGTAACAA
*wgcA*_F	GAACTTGTCAATGCGCCATC
*wgcA*_R	GGCCAGCAATAAATCCTGAATAC
*flaA*_F	CTGGTCACGGCCATCAATAA
*flaA*_R	CAAAGTCCCGCCAGATGTAATA
16S-F3	ATGGCCTTTATGTCCAGGGCTACA
16S-R3	AATCCGAACTACGACGCGCTTTCT
*map*_F	GGACCGGATTTGTCACGATTA
*map*_R	GACGTGGTTGAGGGAAATACA

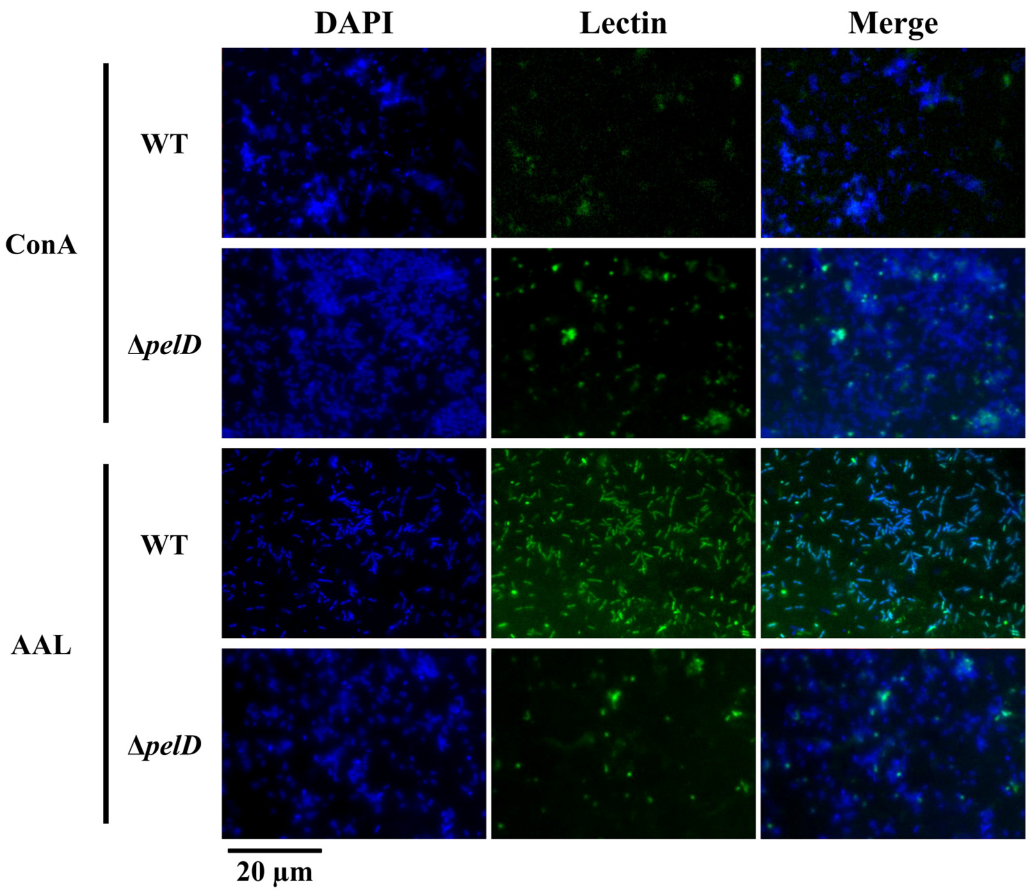

Figure S1. Analysis of PEL exopolysaccharide glycoconjugate composition by using epifluorescence microscopy coupled to FLBA. S°-coupons colonized by *A. thiooxidans*^{T} (WT) or mutant derived (Δ*pelD*) cells were extracted from 5-days growth cultures and incubated with FITC-conjugated AAL or ConA lectins. Then, they were stained with DAPI before microscopy imaging. Size bars represent 20 µm.

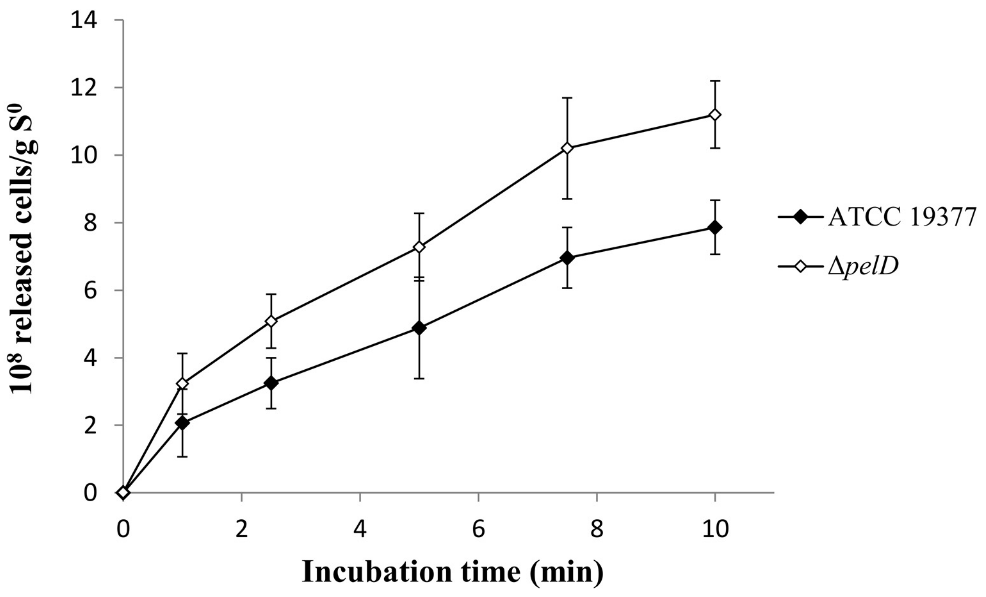

Figure S2. The loss of PEL exopolysaccharide contributes to the production of a fragile biofilm in *A.*

thiooxidans. Inoculated S°-coupons were extracted from 5-days growth cultures and treated with 0.05 % Triton X-100. Then they were vortexed during 10 min. Number of cells released from the wild type and Δ*pelD* null-mutant biofilms subjected to the mechanical stress was determined with a Petroff-Hausser counting chamber and normalized against mass of sulfur. Standard deviations were calculated from three independent cultures.

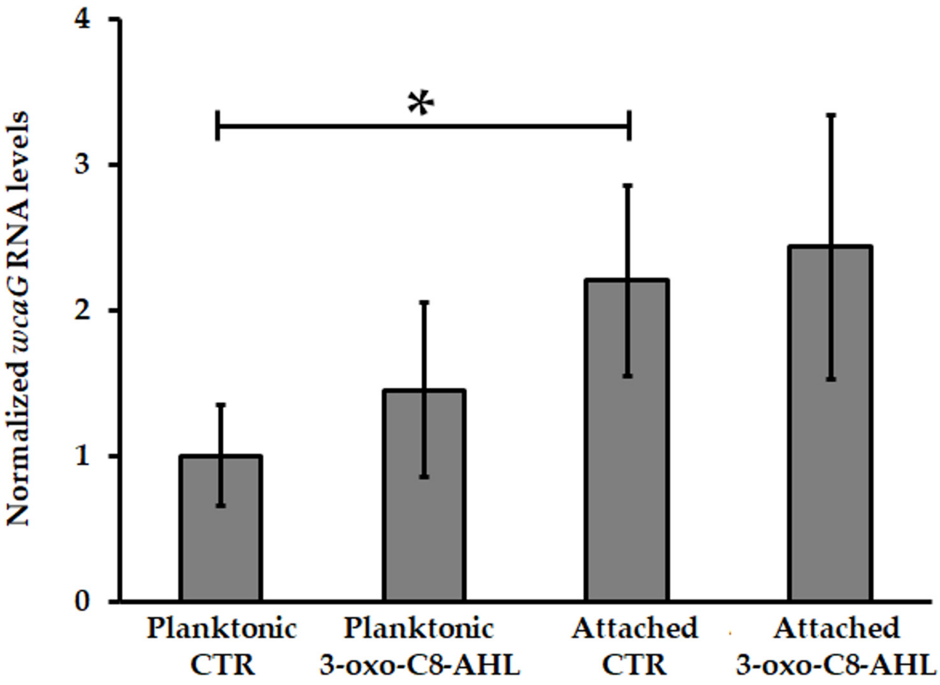

Figure S3. Addition of QS molecule 3-oxo-C8-AHL has no significant effect on transcription levels of *wcaG* from *A. thiooxidans*^{T}. Total RNAs were extracted from *A. thiooxidans* WT cells obtained from 5-days growth cultures. Transcript levels of *wcaG* were measured by qPCR and then normalized using DNA *16S* and *map* genes. Values represent the average of 4 independent experiments ± standard deviation. Significant differences calculated by a one-way ANOVA test ($p < 0.05$) are noted (*). CTR, DMSO 0.01 % without AHL.

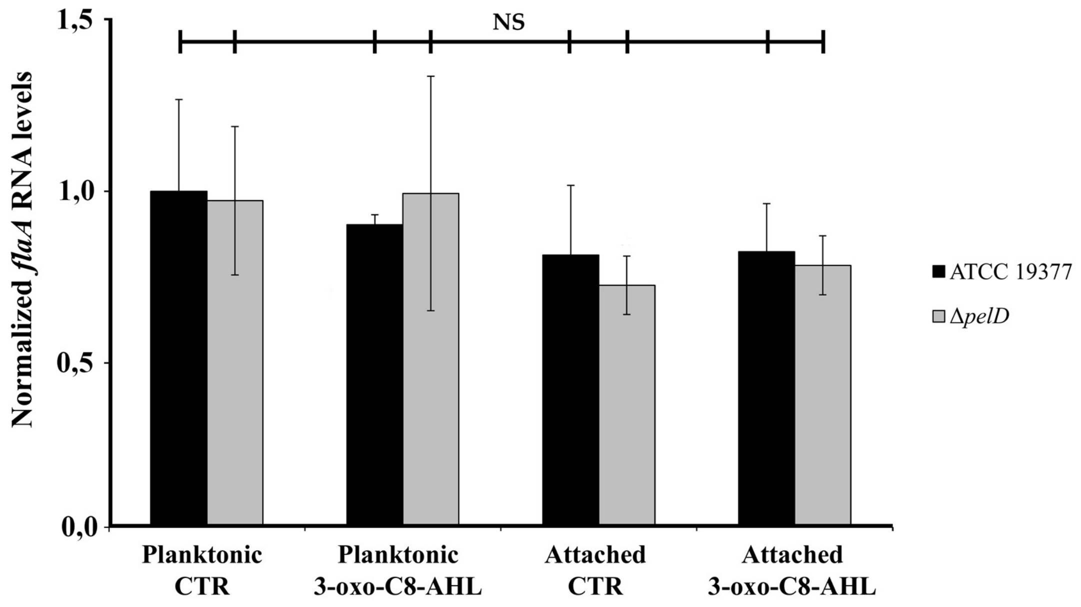

Figure S4. Transcriptional analysis of *flaA* gene from *A. thiooxidans*^{T}. Total RNAs were extracted from *A. thiooxidans* cells obtained from 5-days growth cultures. Transcript levels were measured by qPCR and then normalized using DNA *16S* and *map* genes. Values represent the average of 4 independent experiments ± standard deviation. **NS**, None significant differences were observed by a one-way ANOVA test ($p < 0.05$). CTR, DMSO 0.01 % without AHL.

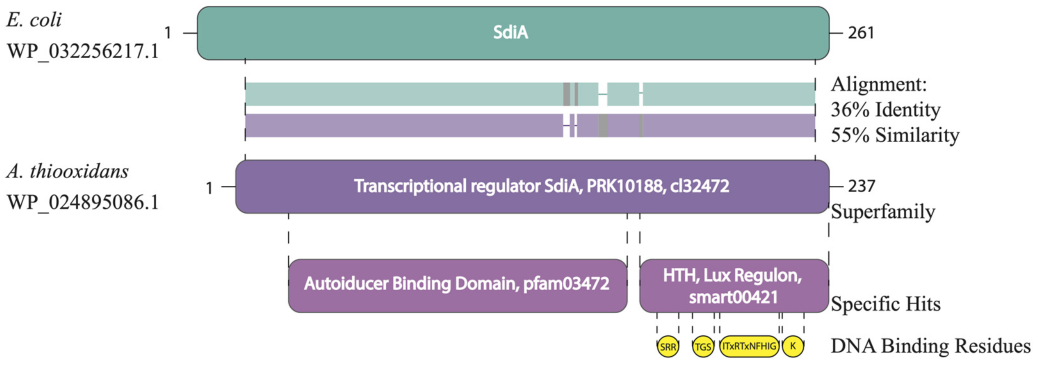

Figure S5. Bioinformatic characterization of WP_024895086.1, a SdiA-like protein present in the new available genome sequences of *A. thiooxidans*. (Top) Schematic representation of SdiA (green) and WP_024895086.1 (purple) amino acid sequence alignment. As noted, both proteins share high similarity and strong identity along over 88% of coverage. Few identified gaps are shown as lines without background color. (Bottom) Domain structure of WP_024895086.1 protein shows the distinctive two domains of canonical QS regulators: Autoinducer Binding Domain (pfam03472) and Helix-Turn-Helix Lux Domain (smart00421), whose combination is classified as PRK10188 Superfamily. Amino acids responsible for DNA binding are depicted in yellow.
